# External transportability of bacterial- and viral-associated host-response modules across public transcriptomic cohorts

**DOI:** 10.1371/journal.pone.0353559

**Published:** 2026-09-10

**Authors:** Reuben S. Maghembe

**Affiliations:** 1 Department of Microbiology and Parasitology, Faculty of Medicine, St. Francis University College of Health and Allied Sciences (SFUCHAS), Ifakara, Tanzania; 2 Department of Omics and Computational Biology, AfroBiomics Co. Ltd., Dar es Salaam, Tanzania; Children's National Hospital, George Washington University, UNITED STATES OF AMERICA

## Abstract

**Objectives:**

To determine whether bacterial- and viral-associated whole-blood host-response modules discovered in GSE211567 retain their prespecified directions when applied unchanged to two external cohorts, including a second cross-platform cohort, and to quantify their robustness to analytical choices and gene deletion.

**Methods:**

Five biologically guided modules were defined and fixed in GSE211567 before external analysis. The same modules were applied unchanged to GSE73461 and GSE72810 using an unweighted mean gene-wise z-score rule. Primary contrasts included 52 bacterial and 94 viral samples in GSE73461 and 23 definite bacterial and 28 definite viral samples in GSE72810. Wilcoxon tests were adjusted across the five modules using the Benjamini-Hochberg method. Hodges-Lehmann shifts, rank-biserial effects and 10,000-replicate bootstrap confidence intervals were calculated. Sensitivity analyses assessed z-score reference populations, probable-case inclusion, probe summarisation, GSVA scoring and exhaustive leave-one/two-gene deletion.

**Results:**

All five modules retained their expected directions in both projection cohorts. BACT_M2, VIR_M1a, VIR_M1b and VIR_M2 had confidence intervals excluding zero and passed false-discovery-rate correction in both cohorts, whereas BACT_M1 remained directionally concordant but borderline in GSE73461 and GSE72810 (adjusted P = 0.0799 in each). All 30 z-reference, case-definition and probe-collapse sensitivity estimates retained the expected direction. Under GSVA, BACT_M1 gained statistical support, whereas VIR_M2 retained its viral-higher direction but lost confidence-interval and adjusted-P-value support. Across 29,826 leave-one/two-gene variants, every variant retained the expected direction and the minimum Pearson correlation with its complete-module score was 0.9940.

**Conclusions:**

The predefined host-response modules retained their expected directions across two external cohorts measured on different Illumina platforms, with the strongest reproducible support for BACT_M2 and the three viral-associated modules. BACT_M1 and the GSVA behaviour of VIR_M2 demonstrate that direction preservation does not imply uniform statistical support across cohorts or scoring algorithms.

## Introduction

Distinguishing bacterial from viral infection remains important for infectious-disease medicine, antimicrobial stewardship and host-response biology [1–4]. Although pathogen detection is central to diagnosis, microbiological results may be delayed, insensitive or difficult to interpret when colonisation, co-infection or previous antimicrobial exposure complicate pathogen attribution [2,5,6]. Host transcriptomic studies have therefore been widely explored as complementary approaches, but much work has emphasised diagnostic classifiers and prediction performance rather than whether the underlying host-response biology is transportable across heterogeneous cohorts [7–10].

A complementary question is whether biologically interpretable response programmes discovered in one setting behave similarly when tested in a different cohort. In this study, transportability means preservation of the prespecified biological direction when the same module definition and scoring rule are applied to another dataset. Fixed-module projection means applying predefined gene sets to an external dataset without changing their genes, expected directions or weights and without retraining a model. This is distinct from diagnostic validation. A classifier may perform well because of cohort-specific, technical or sampling structure, whereas the present analysis asks whether the same biological modules retain their expected bacterial-higher or viral-higher behaviour outside the discovery dataset [8,10,11].

Public infection transcriptomic cohorts are valuable for this purpose, but samples may differ by geography, clinical syndrome, pathogen spectrum, platform, preprocessing and case definition [7,9,11]. Examining the discovery contrast separately within the available geographic strata can reduce the risk that a pooled bacterial-versus-viral signal is driven mainly by one setting. The study therefore kept discovery and external testing separate: module definitions, expected directions and scoring rules were fixed before either external cohort was analysed.

Here, GSE211567 was used to discover bacterial- and viral-associated host-response programmes while requiring cross-site directional support. Five conservative modules were defined before external testing and then applied unchanged to GSE73461 and GSE72810. The latter provided a second accession-level, sample-level and cross-platform cohort. The aim was not to train or validate a diagnostic classifier, but to test whether predefined modules retained their expected directions, quantify cross-cohort effect sizes and determine sensitivity to score reference, probe handling, case definition, alternative gene-set scoring and gene deletion.

## Methods

### Study design and datasets

This study used a staged design that kept discovery separate from external testing. GSE211567 was used for discovery, biological interpretation and definition of the five modules. GSE73461 was analysed only after the module genes, expected directions and scoring rules had been fixed. GSE72810 was subsequently analysed as a second accession-level and sample-level cohort providing cross-platform validation. Neither external dataset was used to reselect genes, rename modules, alter module composition, tune weights or train a diagnostic classifier.

Public host-transcriptomic datasets were considered if they contained infection-relevant human transcriptomic data, recoverable sample metadata, interpretable pathogen-class labels, usable feature identifiers and sufficient sample structure for the intended analysis. GSE161731 was retained only as a technical rehearsal dataset, while GSE261482 and GSE68310 were audited but not selected for formal projection. GSE211567, GSE73461 and GSE72810 were obtained from the Gene Expression Omnibus [12–14].

### GSE211567 discovery and module definition

GSE211567 provided the discovery dataset and contained whole-blood transcriptomic samples from Sri Lanka and the United States [12]. After metadata and expression-quality auditing, the prespecified primary discovery set contained 224 samples: 101 bacterial and 123 viral. The primary limma model evaluated bacterial versus viral infection while adjusting for site and sequencing batch, and separate bacterial-versus-viral models were also fitted within Sri Lanka and the United States. For each modelled feature, directional concordance was defined as agreement in the sign of the bacterial-versus-viral log2 fold change between the contrasts being compared. Percentage directional concordance was the number of same-sign features divided by the number of compared features, multiplied by 100. Spearman correlations of log2 fold changes were used as a complementary measure of ranked effect-size agreement across the pooled and site-specific analyses.

Differential-expression results were treated as a discovery-ranking layer rather than a diagnostic signature [15]. Positive log2 fold-change values were interpreted as bacterial-higher and negative values as viral-higher. Transcript-level features were mapped to gene identifiers, summarised at gene level and carried into Gene Ontology biological-process enrichment [16,17]. Cross-site directionally supported features were prioritised, overlapping or redundant enriched terms were grouped, and candidate biological programmes were reviewed using the documented curation hierarchy. Module selection was therefore a biologically guided and reproducible curation step rather than mathematical optimisation for separation or prediction accuracy. The external-cohort results were not used to choose or modify module genes. Five modules were fixed before external testing: BACT_M1, BACT_M2, VIR_M1a, VIR_M1b and VIR_M2.

### GSE73461 formal external projection

GSE73461 expression, annotation, metadata and group labels were audited independently of module discovery [13]. The cohort was measured on the Illumina GPL10558 platform. The 52 DefiniteBacterial, 94 DefiniteViral and 55 Control samples (n = 201) constituted the main z-score reference population. The primary inferential contrast remained restricted to the 52 DefiniteBacterial versus 94 DefiniteViral samples; Control samples contributed to the reference population but not to the bacterial-versus-viral test. Inflammatory, Kawasaki and Unknown groups were excluded from both the main z-score reference population and the primary contrast.

Illumina probes were mapped to gene symbols, and locked genes were checked against predefined module-coverage thresholds. The numbers of scored genes were 24/25 for BACT_M1, 21/21 for BACT_M2, 128/128 for VIR_M1a, 33/33 for VIR_M1b and 105/106 for VIR_M2.

### GSE72810 cross-platform validation and probe selection

GSE72810 contained 146 paediatric whole-blood samples measured using the Illumina HumanHT-12 v3 platform [14]. All 146 samples were retained in the main z-score reference population, whereas the prespecified primary inferential contrast was restricted to 23 definite bacterial versus 28 definite viral samples. Seventeen probable bacterial and seven probable viral samples were reserved for expanded-case sensitivity analysis. Sixteen controls and 55 uncertain samples contributed to the main z-score reference population but were excluded from the primary bacterial-versus-viral test.

Predefined module genes were reconciled to the GSE72810 platform through Entrez identifiers. Of 313 module-gene instances, 303 were mapped and 10 were unmapped. Module coverage was BACT_M1 24/25, BACT_M2 20/21, VIR_M1a 125/128, VIR_M1b 33/33 and VIR_M2 101/106. When multiple authorised probes represented the same Entrez gene, a prespecified rule selected the probe with the highest median expression across all 146 samples, with lexicographic ordering used only to resolve exact ties. Probe selection was completed before testing group differences.

### Module scoring and statistical analysis

For the primary mean-z analyses, expression for each mapped gene g in sample i was standardised within the prespecified reference population as z_gi = (x_gi – mean_g)/ SD_g. For a module containing K mapped and available genes, the sample-level module score was the unweighted arithmetic mean, score_i = (1/K) sum_g z_gi. Thus, every available gene contributed equally to the primary module score. Missing genes were omitted only after module coverage had been documented. No module was retrained, reweighted or direction-flipped.

Wilcoxon rank-sum tests compared bacterial and viral module scores, with Benjamini-Hochberg correction across the five modules [18]. Positive effects denote bacterial-higher scores and negative effects denote viral-higher scores. In addition to median bacterial-minus-viral differences, the revision analyses calculated Hodges-Lehmann location shifts and rank-biserial effects. Confidence intervals were obtained using 10,000 bootstrap replicates.

### Sensitivity and robustness analyses

Z-reference sensitivity was evaluated by repeating scoring using only the primary bacterial and viral samples as the reference population. GSE72810 sensitivity analyses additionally included probable bacterial and viral cases and compared the locked representative-probe scores with mean scores across all authorised probes. Score concordance was assessed using Pearson and Spearman correlations.

A GSVA sensitivity analysis was performed in GSE73461 using the unchanged module gene sets and both z-reference populations. Because mean-z and GSVA scores have different numerical scales, cross-method interpretation focused on effect direction, rank-biserial effect, confidence intervals and adjusted P values rather than direct comparison of raw score magnitudes.

Gene-deletion robustness was assessed exhaustively in GSE73461 by recalculating every module after removal of each individual gene and every pair of genes. Variant scores were compared with the corresponding complete-module scores, and expected-direction retention, Wilcoxon results and Pearson and Spearman correlations were recorded.

### Cross-cohort interpretation and reproducibility boundaries

The GSE72810 and GSE73461 GEO sample accession sets were disjoint and the cohorts were measured on different Illumina array platforms. However, direct participant overlap could not be assessed because participant identifiers were not deposited, and the studies arose from the same broad investigator network. GSE72810 is therefore described as a second accession-level and sample-level cross-platform cohort rather than as a fully investigator-independent replication cohort.

Analysis scripts, decision logs, quality gates, source-data tables, manuscript-facing figures and supplementary outputs were organised in the project repository. The analyses evaluate transportability and robustness of the predefined biological modules. They do not constitute diagnostic classifier discovery, clinical-performance validation, clinical implementation evidence or causal validation.

### Ethics approval

This study reanalysed publicly available de-identified transcriptomic datasets and did not involve new recruitment of human participants, new collection of human biospecimens or access to identifiable private information. No new ethics approval was required for this secondary analysis of public data.

### Declaration of generative AI and AI-assisted technologies in the manuscript preparation process

During preparation and revision of this manuscript, the author used ChatGPT, an AI-assisted tool provided by OpenAI, to assist with editorial organisation, language refinement, workflow planning, code drafting and checking, formatting checks, and preparation of manuscript and submission-support materials. AI-generated suggestions were not treated as scientific evidence. Analysis scripts were executed against the stated public datasets, and numerical and graphical outputs were checked using the documented reproducibility, source-lock and quality-control procedures. The author reviewed and verified the analysis code, results, references, biological interpretation, figures, tables, manuscript text and submission materials and takes full responsibility for the final work.

## Results

### GSE211567 discovery identifies bacterial- and viral-associated programmes across sites

**Fig 1:**
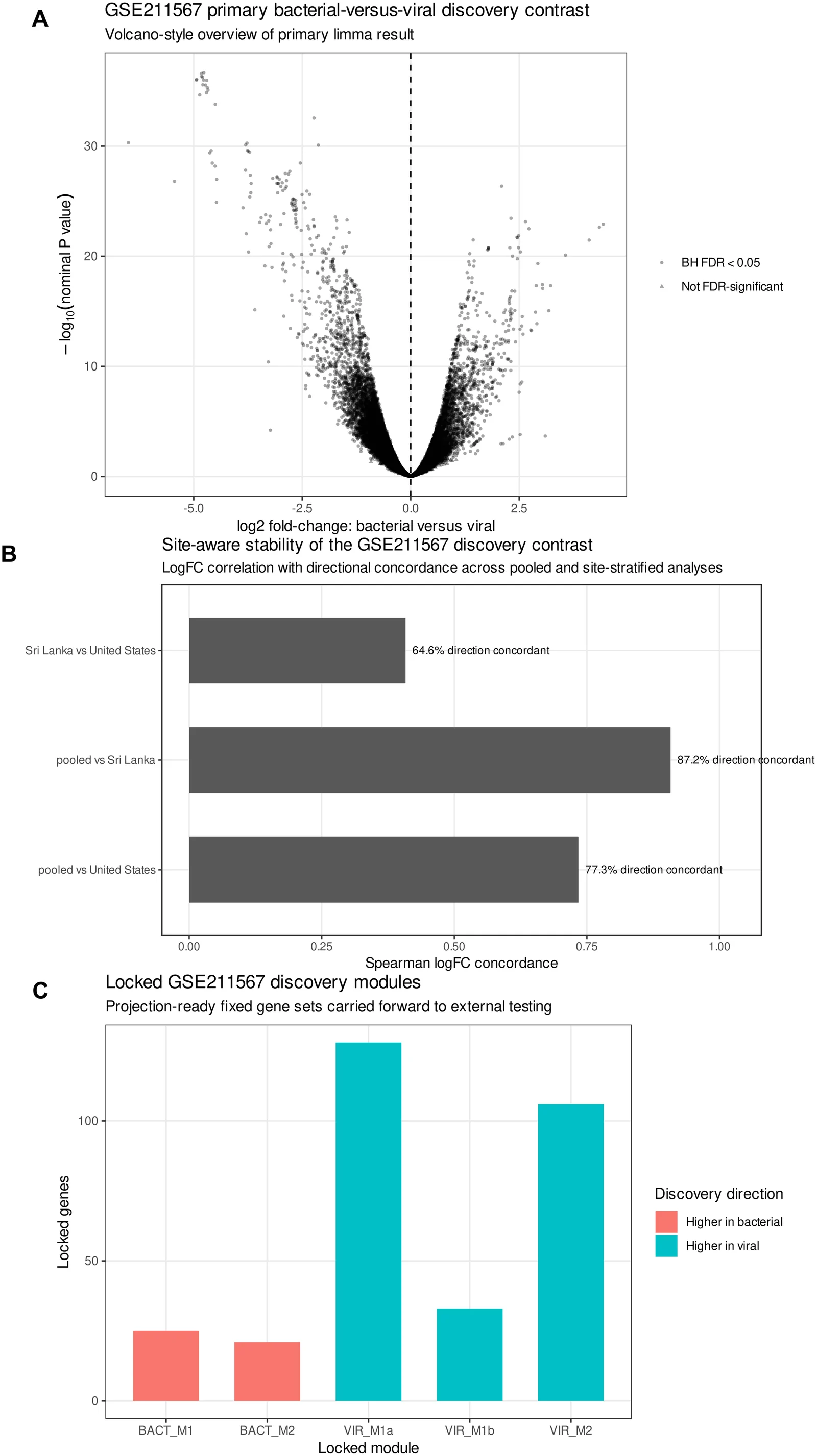
Discovery and conservative definition of bacterial- and viral-associated host-response modules in GSE211567. (A) Primary bacterial-versus-viral discovery analysis in GSE211567. The volcano-style plot summarises the limma-ranked host-transcriptomic contrast used as the discovery starting point before cross-site concordance assessment and biological module definition. Positive log2 fold-change values indicate bacterial-higher features, whereas negative values indicate viral-higher features. (B) Cross-site concordance of the GSE211567 bacterial-versus-viral discovery contrast. Spearman correlations summarise ranked agreement of log2 fold changes between the pooled, Sri Lanka and United States analyses. Directional concordance is the percentage of compared features whose bacterial-versus-viral log2 fold changes have the same sign in the two indicated analyses. This analysis was used to prioritise signals whose direction was not restricted to a single site before pathway interpretation and module definition. (C) The five predefined GSE211567 discovery modules used in external testing. Two modules were bacterial-higher, representing cytoplasmic translation/ribosomal protein activity and mitochondrial respiration/oxidative phosphorylation. Three modules were viral-higher, representing broad antiviral/interferon-stimulated defence, viral restriction/type I interferon signalling and cytokine/innate immune regulation. Module genes and expected directions were fixed before external analysis, with no later gene reselection or module redefinition.

The GSE211567 discovery analysis used a predefined bacterial-versus-viral contrast while keeping module definition separate from external testing. The primary limma analysis ranked host-transcriptomic features before cross-site concordance checks across the pooled and site-stratified analyses (Fig 1A-B). This procedure reduced the likelihood of carrying forward features driven mainly by one geographic or technical stratum.

### Conservative biological curation defines five fixed modules

Directionally eligible features were mapped to genes and assessed using Gene Ontology biological-process enrichment. Redundancy-reduced biological groups were reviewed using the documented curation hierarchy before final module locking. Two modules were bacterial-higher: BACT_M1, representing cytoplasmic translation and ribosomal activity, and BACT_M2, representing mitochondrial respiration and oxidative phosphorylation. Three modules were viral-higher: VIR_M1a, VIR_M1b and VIR_M2, representing broad antiviral/interferon defence, viral restriction/type I interferon activity and cytokine/innate immune regulation, respectively (Fig 1C).

### GSE73461 formal external projection retains all expected directions

The primary GSE73461 contrast contained 52 DefiniteBacterial and 94 DefiniteViral samples. All modules passed the predefined coverage threshold and were scored without gene reselection, module redefinition, reweighting or model training.

**Fig 2:**
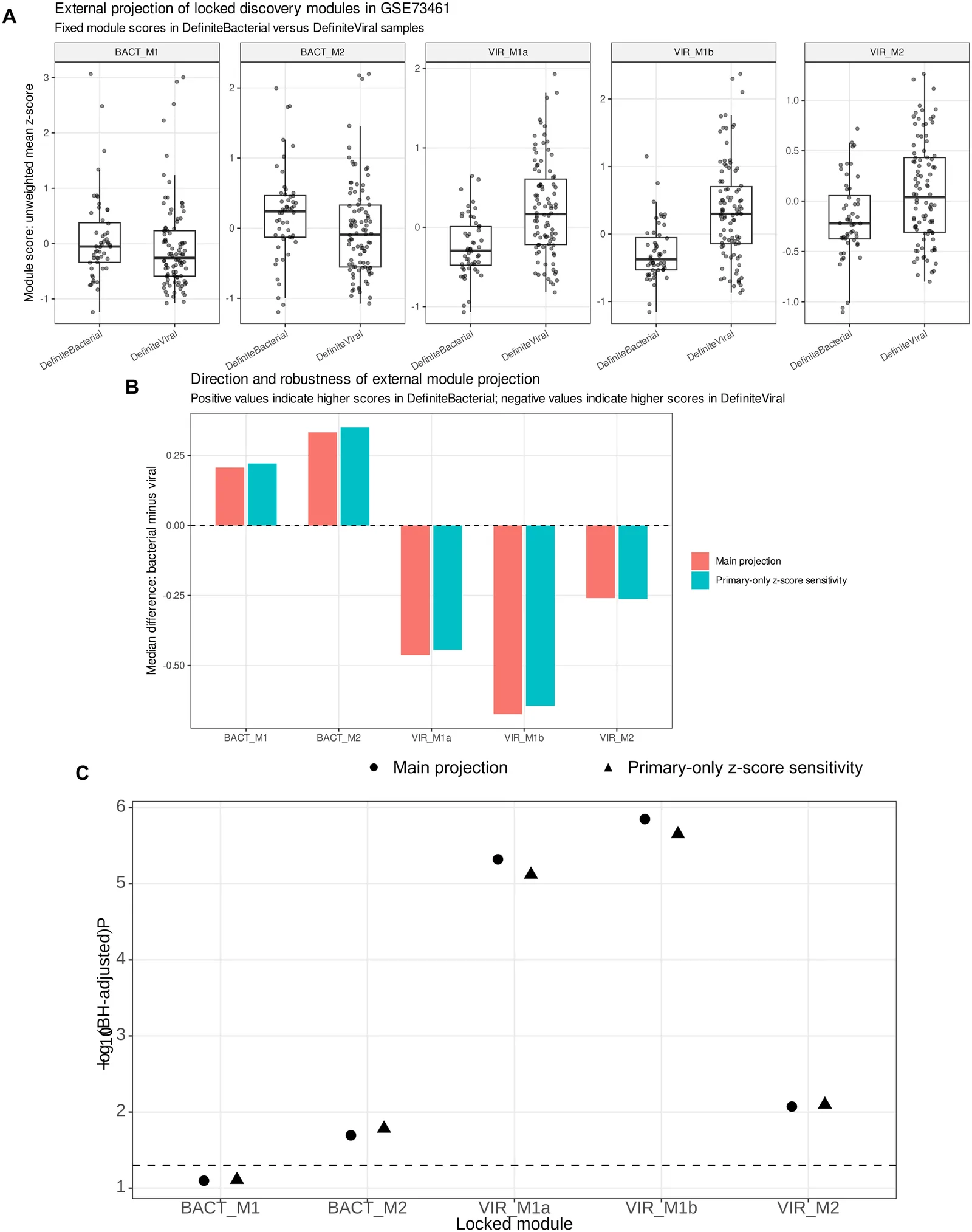
External evaluation of predefined GSE211567 discovery modules in GSE73461. (A) Distribution of locked module scores in the independent GSE73461 external projection cohort. Module scores were calculated using the pre-specified unweighted mean z-score rule in DefiniteBacterial and DefiniteViral samples. Genes were scored without gene reselection, reweighting, module renaming or diagnostic model training. (B) Median bacterial-minus-viral module-score differences in the main GSE73461 projection and in the primary-only z-score sensitivity analysis. Positive values indicate higher scores in DefiniteBacterial samples, whereas negative values indicate higher scores in DefiniteViral samples. All five modules retained the expected discovery direction in both analyses. (C) BH-adjusted Wilcoxon P values for the main projection and the primary-only z-score sensitivity analysis are shown as independent points for each categorical module. Circles denote the main projection and triangles denote the primary-only z-score sensitivity analysis. The dashed horizontal line indicates BH-adjusted P = 0.05. The module categories are not connected because they are distinct predefined gene sets rather than points on a continuous sequence. BACT_M1 remained directionally concordant but borderline, whereas BACT_M2 and all three viral-associated modules passed the adjusted-P-value threshold in both mean-z analyses.

All five modules retained their discovery directions (Fig 2; Table 1). BACT_M2 was bacterial-higher, with a median bacterial-minus-viral score difference of +0.3328 and BH-adjusted Wilcoxon P = 0.0202. BACT_M1 was also bacterial-higher but remained borderline after correction, with a median difference of +0.2067 and adjusted P = 0.0799. VIR_M1a, VIR_M1b and VIR_M2 were viral-higher, with median differences of −0.4629, −0.6739 and −0.2596 and adjusted P values of 4.77 x 10^-6, 1.41 x 10^-6 and 0.00848, respectively. Primary-only z-reference scoring retained all expected directions and the same four-module pattern of adjusted statistical support.

**Table 1:** External evaluation of the five predefined GSE211567 discovery modules in GSE73461.

Module	Biological label	Discovery direction	Locked genes	GSE73461 genes scored	Main projection median difference; BH-adjusted P	Primary-only z-score sensitivity median difference; BH-adjusted P	Expected direction retained (main)	Expected direction retained (sensitivity)	Interpretation	Missing genes
BACT_M1	Bacterial-higher cytoplasmic translation and ribosomal protein programme	Higher in bacterial	25	24	+0.2067; BH P = 0.0799	+0.2211; BH P = 0.0778	Yes	Yes	Directionally concordant but borderline	HYDIN2
BACT_M2	Bacterial-higher mitochondrial respiration and oxidative phosphorylation programme	Higher in bacterial	21	21	+0.3328; BH P = 0.0202	+0.3504; BH P = 0.0165	Yes	Yes	Robustly externally transported	
VIR_M1a	Viral-higher broad antiviral and interferon-stimulated defence programme	Higher in viral	128	128	−0.4629; BH P = 4.77e-06	−0.4441; BH P = 7.57e-06	Yes	Yes	Strongly and robustly externally transported	
VIR_M1b	Viral-higher viral restriction and type I interferon signalling subgroup	Higher in viral	33	33	−0.6739; BH P = 1.41e-06	−0.6445; BH P = 2.22e-06	Yes	Yes	Strongly and robustly externally transported	
VIR_M2	Viral-higher cytokine and innate immune regulation programme	Higher in viral	106	105	−0.2596; BH P = 0.00848	−0.2626; BH P = 0.00796	Yes	Yes	Robustly externally transported	BTN2A3P

Module scores were calculated using the pre-specified unweighted mean z-score rule without gene reselection, module redefinition, reweighting or diagnostic model training. The primary projection contrast compared DefiniteBacterial and DefiniteViral samples.

**Discovery direction:** Direction assigned during GSE211567 discovery-module locking before external projection.

**Locked genes:** Number of genes fixed in the locked GSE211567 discovery module before GSE73461 projection.

**Genes scored in GSE73461:** Number of locked module genes successfully mapped and scored in GSE73461 after Illumina probe annotation.

**Main projection result:** Median bacterial-minus-viral module-score difference and Benjamini–Hochberg adjusted Wilcoxon P value from the main GSE73461 projection analysis.

**Primary-only z-score sensitivity result:** Median bacterial-minus-viral module-score difference and Benjamini–Hochberg adjusted Wilcoxon P value after gene-wise z-scoring using only DefiniteBacterial and DefiniteViral samples, excluding Control samples from the z-score reference set.

**Interpretation tier:** Conservative interpretation based on direction concordance, statistical support and robustness in the primary-only z-score sensitivity analysis.

**Abbreviations:** BH, Benjamini–Hochberg; OXPHOS, oxidative phosphorylation.

Table 1 reports fixed-module external projection. It should not be interpreted as diagnostic classifier discovery, model training, gene rediscovery, module redefinition or causal validation.

### GSE72810 provides accession-level and cross-platform validation

The GSE72810 primary contrast contained 23 definite bacterial and 28 definite viral samples. Coverage of the predefined module genes remained high after Entrez reconciliation and prespecified representative-probe selection. All five modules retained their expected directions.

BACT_M2 had a Hodges-Lehmann bacterial-minus-viral shift of +0.425 (95% CI + 0.161 to +0.676; adjusted P = 0.0020). VIR_M1a, VIR_M1b and VIR_M2 had shifts of −0.726 (95% CI −0.907 to −0.577), −0.941 (95% CI −1.201 to −0.752) and −0.576 (95% CI −0.682 to −0.475), with adjusted P values of 7.16 x 10^-8, 8.77 x 10^-8 and 8.77 x 10^-8, respectively. BACT_M1 was bacterial-higher but borderline, with a shift of +0.266 (95% CI −0.024 to +0.722; adjusted P = 0.0799).

### Cross-cohort effects support four modules in both cohorts

**Fig 3:**
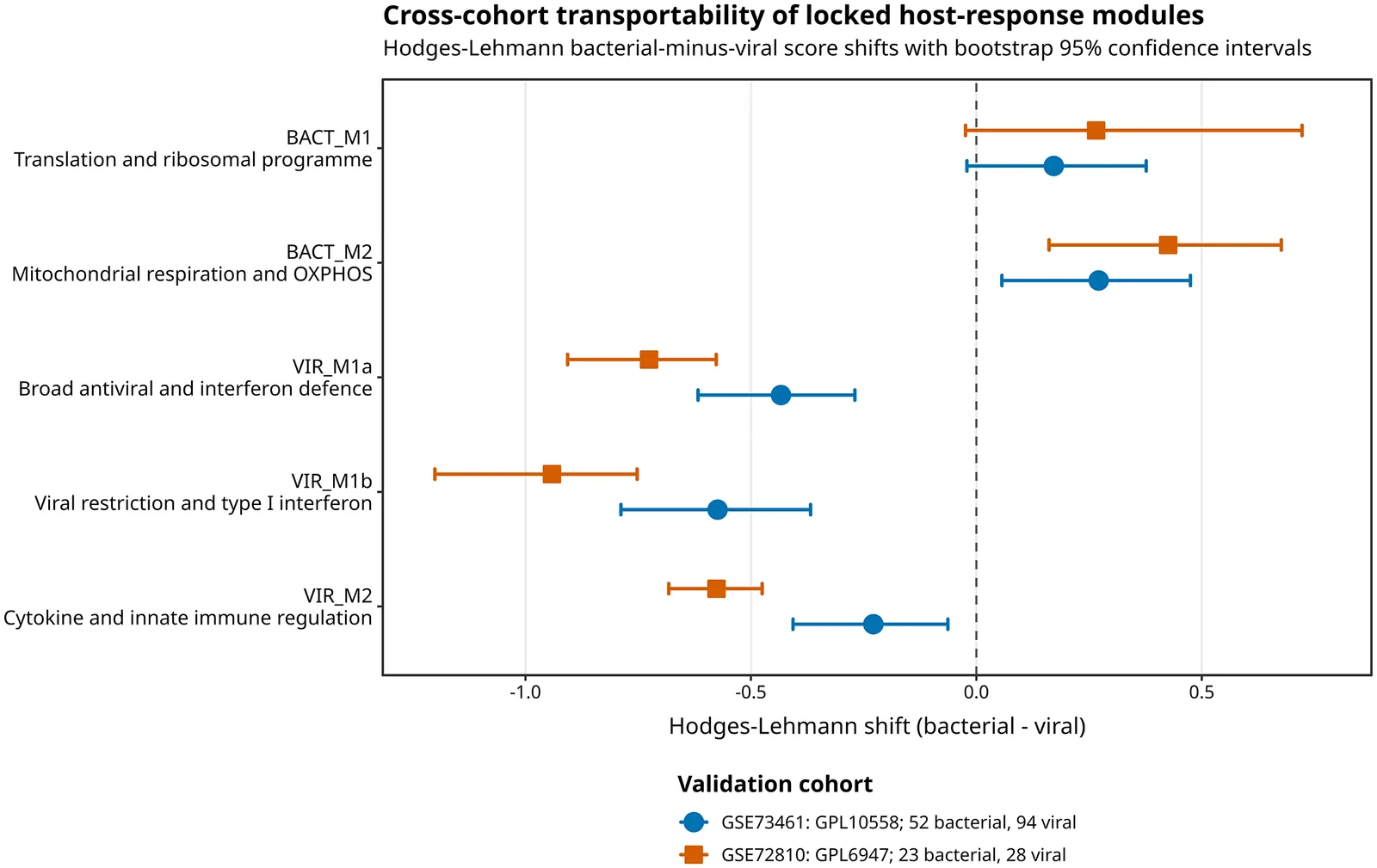
Cross-cohort validation of locked module effects in GSE73461 and GSE72810.

For harmonised cross-cohort comparison, Fig 3 and Table 2 report Hodges-Lehmann bacterial-minus-viral shifts with bootstrap 95% confidence intervals; these estimates are distinct from the median score differences reported for the original GSE73461 projection in Fig 2 and Table 1. All ten cohort-module effects retained the expected direction (Fig 3; Table 2). BACT_M2, VIR_M1a, VIR_M1b and VIR_M2 had confidence intervals excluding zero and passed false-discovery-rate correction in both GSE73461 and GSE72810. BACT_M1 was directionally concordant in both cohorts, but both confidence intervals included zero and both adjusted P values were approximately 0.08.

**Table 2:** Cross-cohort Hodges-Lehmann effects for the five locked host-response modules.

Module	GSE73461 effect (95% CI)	GSE73461 BH-adjusted P	GSE72810 effect (95% CI)	GSE72810 BH-adjusted P	Expected direction retained in both	CI excludes zero in both	FDR significant in both	Interpretation
BACT_M1	+0.172 (95% CI −0.021 to +0.377)	0.0799	+0.266 (95% CI −0.024 to +0.722)	0.0799	Yes	No	No	Directionally concordant in both cohorts but statistically borderline, with confidence intervals including zero.
BACT_M2	+0.271 (95% CI + 0.057 to +0.475)	0.0202	+0.425 (95% CI + 0.161 to +0.676)	0.0020	Yes	Yes	Yes	Expected-direction support with confidence intervals excluding zero in both cohorts.
VIR_M1a	−0.433 (95% CI −0.617 to −0.269)	4.77e-06	−0.726 (95% CI −0.907 to −0.577)	7.16e-08	Yes	Yes	Yes	Strong expected-direction support with confidence intervals excluding zero in both cohorts.
VIR_M1b	−0.574 (95% CI −0.788 to −0.368)	1.41e-06	−0.941 (95% CI −1.201 to −0.752)	8.77e-08	Yes	Yes	Yes	Strong expected-direction support with confidence intervals excluding zero in both cohorts.
VIR_M2	−0.228 (95% CI −0.407 to −0.063)	0.0085	−0.576 (95% CI −0.682 to −0.475)	8.77e-08	Yes	Yes	Yes	Expected-direction support with confidence intervals excluding zero in both cohorts.

Hodges-Lehmann bacterial-minus-viral module-score shifts are shown with stratified nonparametric bootstrap 95% confidence intervals for the five modules predefined in the GSE211567 discovery analysis.

Positive estimates indicate higher module scores in bacterial samples, whereas negative estimates indicate higher scores in viral samples. GSE73461 contained 52 DefiniteBacterial and 94 DefiniteViral samples measured on GPL10558. GSE72810 contained 23 definite bacterial and 28 definite viral samples measured on GPL6947.

BACT_M2, VIR_M1a, VIR_M1b and VIR_M2 retained their expected directions with confidence intervals excluding zero and BH-adjusted Wilcoxon P values below 0.05 in both cohorts. BACT_M1 retained the expected bacterial-higher direction in both cohorts but remained statistically borderline.

The modules were scored without gene reselection, module redefinition, gene reweighting, direction flipping or diagnostic-model training.

GSE72810 was analysed as a second accession-level and sample-level cohort providing cross-platform validation. Its GSM accession set was disjoint from GSE73461 and it used a different Illumina platform. Direct participant overlap could not be assessed because participant identifiers were not deposited, and the studies arose from the same broad investigator network.

Effects are Hodges-Lehmann bacterial-minus-viral location shifts with bootstrap 95% confidence intervals. Positive values indicate bacterial-higher scores and negative values indicate viral-higher scores. Benjamini-Hochberg adjustment was applied across the five modules within each cohort.

The cross-cohort pattern therefore supported four modules under both effect-size and adjusted-significance criteria. The strongest and most consistent signals were the viral-associated modules, followed by the bacterial mitochondrial respiration/OXPHOS module.

### Sensitivity analyses retain direction but identify method-dependent support

Across the two GSE73461 z-reference analyses and four GSE72810 z-reference, case-definition and probe-collapse analyses, all 30 module estimates retained the expected direction (S1 Fig, panel A). Twenty-four estimates passed false-discovery-rate correction and 25 rank-biserial confidence intervals excluded zero. GSE72810 score representations were highly concordant; the minimum Pearson correlation was 0.9874 and the minimum Spearman correlation was 0.9814.

The GSVA analysis retained all expected directions but showed non-uniform inferential support (S1 Fig, panel B). BACT_M2, VIR_M1a and VIR_M1b remained supported under both mean-z and GSVA scoring. BACT_M1 was borderline under mean-z scoring but gained confidence-interval and adjusted-P-value support under GSVA. In contrast, VIR_M2 was supported under mean-z scoring but was near zero and statistically unsupported under GSVA in both reference populations. VIR_M2 is therefore scoring-method-sensitive rather than uniformly robust across algorithms.

### Exhaustive deletion analysis supports distributed module signal

The leave-one/two-gene analysis evaluated 29,826 module variants across the two GSE73461 scoring populations (S1 Fig, panel C). Every variant retained the expected module direction. The minimum Pearson correlation with the corresponding complete-module score was 0.9940. These results indicate that the observed module behaviour was not driven by removal-sensitive dependence on one gene or one gene pair, while not establishing causal sufficiency of individual genes.

## Discussion

This study evaluated whether biologically curated whole-blood host-response modules discovered in GSE211567 retained their prespecified bacterial-higher or viral-higher behaviour when the same definitions and scoring rules were applied to two external cohorts. All five modules retained their expected directions in both GSE73461 and GSE72810. Four modules – BACT_M2, VIR_M1a, VIR_M1b and VIR_M2 - had confidence intervals excluding zero and passed adjusted-significance thresholds in both cohorts. BACT_M1 was directionally concordant but borderline in each cohort, indicating a reproducible direction with less stable inferential support.

The viral-associated modules showed the strongest transportability. VIR_M1a and VIR_M1b captured broad antiviral, interferon-stimulated and viral-restriction programmes, while VIR_M2 represented cytokine and innate immune regulation. Their cross-cohort behaviour is consistent with the central role of interferon-linked responses in viral infection and with prior studies in which interferon-inducible biomarkers contributed to viral-versus-bacterial discrimination [3,4,19]. BACT_M2 also transported consistently, supporting a bacterial-associated mitochondrial respiration and oxidative-phosphorylation programme in these datasets and aligning with evidence that inflammatory activation can remodel leukocyte immunometabolism [20,21].

The sensitivity analyses refine rather than uniformly strengthen this interpretation. Direction was stable across z-reference populations, GSE72810 case definitions and probe handling, and GSE72810 score correlations remained high. Exhaustive deletion analysis also supported a distributed module signal. However, GSVA changed inferential support for two modules: BACT_M1 became supported, whereas VIR_M2 lost statistical support despite retaining the expected sign. Directional robustness, effect-size robustness and statistical robustness should therefore be reported as related but distinct properties.

The principal contribution is not a diagnostic classifier, but a transparent framework for asking whether predefined biological modules retain their behaviour in new datasets. Fixing the module genes, expected directions and scoring rules before external testing, and prohibiting gene reselection, sign reversal, reweighting and model retraining, reduces rediscovery bias. Hodges-Lehmann shifts, rank-biserial effects, confidence intervals, cross-platform testing and deletion analyses provide complementary evidence about the magnitude and stability of the observed programmes.

Several limitations remain. Public metadata and infection adjudication were imperfect, and the cohorts differed in age, syndrome, pathogen composition, platform and preprocessing. Whole-blood scores may reflect both cell-composition changes and cell-intrinsic activation. The GSE72810 and GSE73461 GEO sample accession sets were disjoint and were measured on different Illumina platforms, but direct participant overlap could not be assessed because participant identifiers were not deposited; the studies also arose from the same broad investigator network. GSE72810 should therefore not be treated as a fully investigator-independent replication cohort. The retrospective analyses do not establish clinical performance, clinical readiness or causal mechanisms. Prospective, investigator-independent and multi-cohort studies are required to determine how these modules behave across age groups, syndromes, pathogens, sampling times and treatment contexts.

### Transparency declaration

#### Code availability.

Analysis scripts, decision logs, quality gates, source-data tables, manuscript-facing figures and supplementary outputs are available in the public project repository at https://github.com/rmaghembe1/host-response-module-transportability. The revision-round code and outputs used for this resubmission are available on the public plosone_revision_round1_2026 branch at https://github.com/rmaghembe1/host-response-module-transportability/tree/plosone_revision_round1_2026.

## Supporting information

S1 Dataset Supplementary Tables S1–S5. External-cohort search register, locked GSE211567 module definitions, GSE73461 identifier coverage and probe choices, GSE73461 sample-level scores, and complete GSE73461 primary and z-reference sensitivity statistics.
(XLSX)

S2 Dataset Supplementary Table S6. GSE72810 cohort audit, sample-classification framework, locked primary and expanded contrasts, and cross-cohort independence assessment.
(XLSX)

S3 Dataset Supplementary Table S7. GSE72810 Entrez reconciliation, module coverage, complete module-gene mapping, and prespecified representative-probe choices.
(XLSX)

S4 Dataset Supplementary Table S8. Privacy-safe GSE72810 sample-level module scores using anonymized analysis identifiers, primary and sensitivity tests, bootstrap effect-size confidence intervals, and score-concordance analyses.
(XLSX)

S5 Dataset Supplementary Table S9. Harmonised GSE73461–GSE72810 cross-cohort effect-size source data and five-module summary.
(XLSX)

S6 Dataset Supplementary Table S10. GSE73461 mean-z/GSVA comparison, score concordance, module-level deletion summary, worst-case deletion variants, and complete exhaustive leave-one/two-gene results.
(XLSX)

S1 Fig Sensitivity and robustness of locked host-response modules. **A, Z-reference, case-definition and probe-collapse sensitivities.** Rank-biserial bacterial-versus-viral effects and bootstrap 95% confidence intervals are shown for the two GSE73461 mean-z analyses and four GSE72810 analyses. All 30 cohort-module estimates retained the expected direction. GSE72810 score concordance across the tested z-reference and probe-collapse representations remained high, with minimum Pearson r = 0.9874 and minimum Spearman rho = 0.9814. **B, Mean-z versus GSVA scoring-method sensitivity in GSE73461.** Rank-biserial effects are displayed because they are comparable across scoring methods with different raw score scales. BACT_M2, VIR_M1a and VIR_M1b retained confidence-interval and BH-adjusted statistical support under both methods and z-reference populations. BACT_M1 was borderline under mean-z scoring but supported under GSVA. VIR_M2 retained a small viral-higher direction under GSVA but its confidence intervals included zero and its BH-adjusted P values were not significant. VIR_M2 is therefore described as scoring-method-sensitive. **C, Exhaustive leave-one/two-gene robustness in GSE73461.** Points show the minimum Pearson correlation between each deletion variant and the corresponding complete-module score. Across 29,826 variants, every leave-one and leave-two analysis retained the expected module direction. The minimum Pearson correlation was 0.9940 and the minimum Spearman correlation was 0.9897. Positive rank-biserial effects indicate bacterial-higher scores and negative effects indicate viral-higher scores. These analyses evaluate robustness of the predefined modules and do not constitute gene reselection, module redefinition, diagnostic-model training or causal validation.
(PNG)
