# Supplementary material for: External transportability of bacterial- and viral-associated host-response modules across public transcriptomic cohorts
Source: PLoS One. 2026 Sep 10;21(9):e0353559. doi: 10.1371/journal.pone.0353559 (PMC13561310; doi:10.1371/journal.pone.0353559)
Supplement: Supplementary file 1 [file pone.0353559.s009.docx]

**Response to Academic Editor and Reviewers**

**Manuscript ID: PONE-D-26-30583**

*External transportability of bacterial- and viral-associated host-response modules across public transcriptomic cohorts*

Reuben S. Maghembe

8 August 2026

Dear Academic Editor and Reviewers,

Thank you for the careful evaluation of the manuscript and for the constructive recommendations. The manuscript has been revised substantially in response. The principal changes include addition of a second accession-level and cross-platform projection cohort (GSE72810), harmonised effect-size and confidence-interval reporting, GSVA sensitivity analysis, exhaustive leave-one/two-gene robustness analysis, expanded cohort and statistical-method descriptions, a revised categorical Figure 2C, clearer definitions and simpler terminology, direct public-data links, public code availability, and a dedicated generative-AI disclosure within Methods.

Each comment is reproduced below, followed by the response and the location of the corresponding revision. The revised submission includes separate clean and marked-up manuscript files.

**Academic Editor / Journal Requirements**

**J1. PLOS ONE style and revision-package requirements**

**Comment:** Ensure PLOS ONE style requirements and file naming are followed.

**Response:** Thank you. The revision package has now been prepared in PLOS ONE submission form. Separate clean and marked-up manuscript files have been generated from the same locked scientific source and subjected to structural, rendering and visual quality-control checks. Continuous line numbering is retained, the two wide tables remain editable and readable in landscape sections, and the marked-up file uses genuine tracked revisions while accepting those revisions reproduces the clean manuscript content.

**Changes in the revised manuscript:** *Final revision package: Manuscript_clean.docx and Manuscript_marked.docx.*

**Supporting analysis/material:** Clean manuscript and marked-up manuscript passed the final DOCX structural and visual QA gates.

**J2. Generative-AI disclosure**

**Comment:** Provide a dedicated generative-AI disclosure naming the tool, describing use, validation of outputs, and affected study/article materials.

**Response:** The manuscript now contains a dedicated generative-AI disclosure within the Methods section. The disclosure identifies ChatGPT as an AI-assisted tool provided by OpenAI, describes its use for editorial organisation, language refinement, workflow planning, code drafting and checking, formatting checks and preparation of submission-support materials, states that AI-generated suggestions were not treated as scientific evidence, describes verification of numerical and graphical outputs, and states that the author reviewed the analysis, references, interpretation, figures, tables and manuscript and takes responsibility for the final work.

**Changes in the revised manuscript:** *Methods, 'Declaration of generative AI and AI-assisted technologies in the manuscript preparation process', immediately before the main Results section.*

**Supporting analysis/material:** The declaration wording was preserved during relocation into Methods; clean and marked-up DOCX copies were synchronised and visually checked.

**J3. Public availability of author-generated code**

**Comment:** Make author-generated code publicly available without restriction.

**Response:** The author-generated analysis code and reproducibility materials are publicly available without access restriction in the project repository. The revision branch contains the revision-round scripts, decision logs, quality gates, source-data tables, manuscript-facing figures and supplementary outputs used to support the revised analyses.

**Changes in the revised manuscript:** *Code availability section.*

**Supporting analysis/material:** Public repository: https://github.com/rmaghembe1/host-response-module-transportability; revision branch: https://github.com/rmaghembe1/host-response-module-transportability/tree/plosone_revision_round1_2026.

**J4. Direct database links in Data Availability**

**Comment:** Provide direct links to each database in the Data Availability Statement.

**Response:** The Data Availability Statement has been revised to provide direct NCBI Gene Expression Omnibus links for each of the three principal datasets: GSE211567, GSE73461 and GSE72810. The text also identifies candidate or technical-rehearsal datasets considered during workflow development.

**Changes in the revised manuscript:** *Data availability section.*

**Supporting analysis/material:** Direct GEO accession URLs for GSE211567, GSE73461 and GSE72810 are included in the revised manuscript.

**Reviewer 1**

**Reviewer 1, Comment 1 — Additional external cohort and limitation**

**Comment:** The conclusions relied on one external validation dataset; include additional datasets or discuss/soften this limitation.

**Response:** We agree that reliance on a single projection cohort was an important limitation. We therefore added GSE72810 as a second accession-level and sample-level whole-blood cohort measured on a different Illumina platform (GPL6947). The cohort contains 146 samples; the prespecified primary contrast comprises 23 definite bacterial and 28 definite viral samples. All five modules retained their expected directions. BACT_M2, VIR_M1a, VIR_M1b and VIR_M2 had confidence intervals excluding zero and passed false-discovery-rate correction in both GSE73461 and GSE72810, whereas BACT_M1 remained directionally concordant but borderline in both. We also softened the replication language because the two GEO accession sets were disjoint and used different array platforms, but direct participant overlap could not be assessed and the studies arose from the same broad investigator network.

**Changes in the revised manuscript:** *Methods: 'GSE72810 cross-platform validation and probe selection' and 'Cross-cohort interpretation and reproducibility boundaries'; Results: 'GSE72810 provides accession-level and cross-platform validation' and 'Cross-cohort effects support four modules in both cohorts'; Discussion limitations.*

**Supporting analysis/material:** Figure 3; Table 2; Supplementary Tables S6-S9; GSE72810 primary Hodges-Lehmann shifts and bootstrap confidence intervals.

**Reviewer 1, Comment 2 — Manual GO review and module definition**

**Comment:** Provide more detail on criteria used during manual GO review/module definition and clarify whether the modules are optimal or most informative.

**Response:** We expanded the Methods to make the module-definition procedure more transparent. Directionally supported discovery features were mapped to genes, assessed by Gene Ontology biological-process enrichment, and reviewed after grouping overlapping or redundant enriched terms. Candidate programmes were evaluated using the documented biological curation hierarchy. We now state explicitly that this was a biologically guided, reproducible curation step rather than mathematical optimisation for group separation or prediction accuracy. External-cohort results were not used to choose or modify module genes.

**Changes in the revised manuscript:** *Methods, 'GSE211567 discovery and module definition'.*

**Supporting analysis/material:** Locked module definitions are unchanged; the revision clarifies the selection rationale and its non-optimisation character.

**Reviewer 1, Comment 3 — Comparison with GSVA**

**Comment:** Compare the proposed scoring strategy with GSVA.

**Response:** We added a GSVA sensitivity analysis in GSE73461 using the same unchanged module gene sets and both z-reference populations. BACT_M2, VIR_M1a and VIR_M1b retained statistical support under both mean-z and GSVA scoring. BACT_M1 was borderline under mean-z scoring but gained statistical support under GSVA. VIR_M2 retained its expected viral-higher direction but became near-zero and statistically unsupported under GSVA. The revision therefore distinguishes direction preservation from method-independent inferential support and describes VIR_M2 as scoring-method-sensitive.

**Changes in the revised manuscript:** *Methods, 'Sensitivity and robustness analyses'; Results, 'Sensitivity analyses retain direction but identify method-dependent support'; Discussion.*

**Supporting analysis/material:** S1 Fig panel B and Supplementary Table S10.

**Reviewer 1, Comment 4 — Effect sizes and confidence intervals**

**Comment:** Report effect sizes and confidence intervals in addition to medians and adjusted P values.

**Response:** We added effect-size estimates and uncertainty intervals throughout the external-cohort analyses. The revised Methods specify Hodges-Lehmann location shifts, rank-biserial effects and stratified 10,000-replicate bootstrap 95% confidence intervals. Figure 3 and Table 2 provide harmonised cross-cohort Hodges-Lehmann effects and 95% confidence intervals for all five modules. This allows the magnitude and uncertainty of the observed effects to be assessed separately from adjusted P values.

**Changes in the revised manuscript:** *Methods, 'Module scoring and statistical analysis'; Results, GSE72810 and cross-cohort effect sections; Figure 3; Table 2.*

**Supporting analysis/material:** Supplementary Tables S8-S9 and cross-cohort Figure 3 source data.

**Reviewer 2**

**Reviewer 2, Comment 1 — Leave-one/two-gene robustness**

**Comment:** Evaluate how loss of specific genes affects module discrimination, for example leave-one-out and leave-two-out analyses.

**Response:** We performed exhaustive leave-one-gene and leave-two-gene sensitivity analyses for every module under both GSE73461 scoring-reference populations. In total, 29,826 module variants were evaluated. Every variant retained the expected module direction. The minimum Pearson correlation between a deletion variant and its corresponding complete module score was 0.9940, and the minimum Spearman correlation was 0.9897. These results support a distributed module signal rather than dependence on removal-sensitive individual genes or gene pairs.

**Changes in the revised manuscript:** *Methods, 'Sensitivity and robustness analyses'; Results, 'Exhaustive deletion analysis supports distributed module signal'.*

**Supporting analysis/material:** S1 Fig panel C; Supplementary Table S10, including complete leave-one/two-gene results.

**Reviewer 2, Comment 2 — Dataset description**

**Comment:** Describe the nature and sample composition of the analyzed datasets in substantially greater detail.

**Response:** The dataset descriptions have been substantially expanded. GSE211567 is identified as whole-blood transcriptomic data from Sri Lanka and the United States, with a prespecified discovery set of 224 samples (101 bacterial and 123 viral). GSE73461 is described with its GPL10558 platform, 52 definite bacterial, 94 definite viral and 55 control samples in the main z-score reference population, and the restricted primary inferential contrast. GSE72810 is described as 146 paediatric whole-blood samples on GPL6947, including definite, probable, control and uncertain groups and the prespecified 23-versus-28 primary contrast.

**Changes in the revised manuscript:** *Methods, 'Study design and datasets', 'GSE211567 discovery and module definition', 'GSE73461 formal external projection', and 'GSE72810 cross-platform validation and probe selection'.*

**Supporting analysis/material:** Supplementary cohort-audit and sample-classification tables.

**Reviewer 2, Comment 3 — Control samples and z-score reference**

**Comment:** Explain the logic and consequences of control-sample inclusion/exclusion in the z-score reference population.

**Response:** We clarified the role of control and non-primary samples in score standardisation versus inference. In GSE73461, the 52 definite bacterial, 94 definite viral and 55 control samples constitute the main gene-wise z-score reference population, but control samples are not included in the bacterial-versus-viral inferential test. A primary-only z-reference sensitivity analysis quantifies dependence on that choice. In GSE72810, all 146 samples contribute to the main z-score reference population, whereas the primary inferential contrast remains restricted to the 23 definite bacterial and 28 definite viral samples. Additional case-definition and probe-collapse analyses test the robustness of these choices.

**Changes in the revised manuscript:** *Methods, 'GSE73461 formal external projection', 'GSE72810 cross-platform validation and probe selection', and 'Sensitivity and robustness analyses'.*

**Supporting analysis/material:** Figure 2, S1 Fig panel A and the supplementary sensitivity tables.

**Reviewer 2, Comment 4 — Figure captions**

**Comment:** Rewrite figure captions so they explain the finding, analysis and figure elements.

**Response:** All principal figure legends were rewritten so that they explain both the analysis and the graphical elements. The revised legends define direction conventions, sample groups, symbols, confidence intervals or adjusted-P-value thresholds where applicable, and state the principal finding represented in each panel. The S1 Fig legend likewise explains the z-reference/case-definition/probe-collapse sensitivity analyses, mean-z versus GSVA comparison and exhaustive gene-deletion analysis.

**Changes in the revised manuscript:** *Legends for Figures 1-3 and Supporting Information S1 Fig.*

**Supporting analysis/material:** Revised manuscript figure captions and supporting-information caption.

**Reviewer 2, Comment 5 — Formal concordance definition**

**Comment:** Provide the formal definition of concordance used in Figure 1B.

**Response:** We now define directional concordance formally. For each modelled feature, concordance means agreement in the sign of the bacterial-versus-viral log2 fold change between the two contrasts being compared. Percentage directional concordance is the number of same-sign features divided by the number of compared features and multiplied by 100. Spearman correlations of log2 fold changes are reported separately as a complementary measure of ranked effect-size agreement.

**Changes in the revised manuscript:** *Methods, 'GSE211567 discovery and module definition'; Figure 1B legend.*

**Supporting analysis/material:** The definition now matches the implemented cross-site concordance calculation.

**Reviewer 2, Comment 6 — Module-score definition**

**Comment:** Define the module score and explain what the mean z-score represents.

**Response:** We added the explicit module-scoring equations. For mapped gene g in sample i, expression is standardised within the prespecified reference population as z_gi = (x_gi - mean_g) / SD_g. For a module with K mapped and available genes, the sample-level score is the unweighted arithmetic mean, score_i = (1/K) sum_g z_gi. Thus each available mapped gene contributes equally to the primary score; missing genes are omitted only after module coverage has been documented.

**Changes in the revised manuscript:** *Methods, 'Module scoring and statistical analysis'.*

**Supporting analysis/material:** The explicit equations and interpretation of the mean z-score are now given in the manuscript.

**Reviewer 2, Comment 7 — Figure 2C categorical display**

**Comment:** Clarify Figure 2C and remove inappropriate lines connecting categorical module values.

**Response:** Figure 2C was rebuilt to treat the module categories correctly as independent categorical values. Circles denote the main projection and triangles denote the primary-only z-score sensitivity analysis. The module categories are not connected by lines. The dashed horizontal line marks BH-adjusted P = 0.05. The revised caption explains each symbol and why no connecting line is used.

**Changes in the revised manuscript:** *Figure 2C and Figure 2 legend.*

**Supporting analysis/material:** Revised Figure 2C generated by the reproducible figure script; publication outputs include PNG and editable SVG versions.

**Reviewer 2 — Statistical-analysis description**

**Comment:** Improve description of statistical analyses.

**Response:** The statistical-analysis description has been expanded substantially. The revised Methods specify Wilcoxon rank-sum testing, Benjamini-Hochberg correction across the five modules, the interpretation of positive and negative effects, median bacterial-minus-viral differences, Hodges-Lehmann shifts, rank-biserial effects, 10,000-replicate bootstrap confidence intervals, Pearson and Spearman score correlations, and the interpretive basis for cross-method comparisons in the GSVA sensitivity analysis.

**Changes in the revised manuscript:** *Methods, 'Module scoring and statistical analysis' and 'Sensitivity and robustness analyses'.*

**Supporting analysis/material:** Supplementary statistical outputs provide the corresponding complete module-level results.

**Reviewer 2 — Clarity and terminology**

**Comment:** Use simpler English; define transportability and fixed-module projection and reduce jargon such as site-aware, safeguards, firewall and frozen.

**Response:** The manuscript was edited throughout for simpler and more precise wording. The title was reframed around external transportability. The Introduction now defines transportability as preservation of the prespecified biological direction when the same module definition and scoring rule are applied to another dataset, and defines fixed-module projection as applying predefined gene sets without changing their genes, expected directions or weights and without retraining a model. Workflow-oriented terms such as 'firewall', 'frozen', 'safeguards' and similar jargon were removed from manuscript-facing prose.

**Changes in the revised manuscript:** *Title; Abstract; Introduction; Methods; Results; Discussion; figure captions throughout the revised manuscript.*

**Supporting analysis/material:** The revised manuscript consistently distinguishes biological transportability from diagnostic-classifier validation.

**Closing statement**

We appreciate the editor's and reviewers' comments, which led to substantial improvements in the analytical robustness, transparency and clarity of the manuscript. All 17 editor/reviewer response items represented in the revision matrix are addressed in the revised manuscript and supporting materials.

**Sincerely,
Reuben S. Maghembe**
