# Supplementary material for: External transportability of bacterial- and viral-associated host-response modules across public transcriptomic cohorts
Source: PLoS One. 2026 Sep 10;21(9):e0353559. doi: 10.1371/journal.pone.0353559 (PMC13561310; doi:10.1371/journal.pone.0353559)
Supplement: Peer review history

# Peer review: Decision Letter 0

**DOI:** 10.1371/journal.pone.0353559.r001

**Authors:** Guichuan Lai

20 Jul 2026

Dear Dr. Maghembe,

Thank you for submitting your manuscript to PLOS ONE. After careful consideration, we feel that it has merit but does not fully meet PLOS ONE’s publication criteria as it currently stands. Therefore, we invite you to submit a revised version of the manuscript that addresses the points raised during the review process.

Please submit your revised manuscript by Sep 03 2026 11:59PM. If you will need more time than this to complete your revisions, please reply to this message or contact the journal office at plosone@plos.org. When you're ready to submit your revision, log on to https://www.editorialmanager.com/pone/ and select the 'Submissions Needing Revision' folder to locate your manuscript file.

- A letter that responds to each point raised by the academic editor and reviewer(s). You should upload this letter as a separate file labeled 'Response to Reviewers'.
- A marked-up copy of your manuscript that highlights changes made to the original version. You should upload this as a separate file labeled 'Revised Manuscript with Track Changes'.
- An unmarked version of your revised paper without tracked changes. You should upload this as a separate file labeled 'Manuscript'.

If you would like to make changes to your financial disclosure, please include your updated statement in your cover letter. Guidelines for resubmitting your figure files are available below the reviewer comments at the end of this letter.

If applicable, we recommend that you deposit your laboratory protocols in protocols.io to enhance the reproducibility of your results. Protocols.io assigns your protocol its own identifier (DOI) so that it can be cited independently in the future. For instructions see: https://journals.plos.org/plosone/s/submission-guidelines#loc-laboratory-protocols. Additionally, PLOS ONE offers an option for publishing peer-reviewed Lab Protocol articles, which describe protocols hosted on protocols.io. Read more information on sharing protocols at https://plos.org/protocols?utm_medium=editorial-email&utm_source=authorletters&utm_campaign=protocols.

As the corresponding author, your ORCID iD is verified in the submission system and will appear in the published article. PLOS supports the use of ORCID, and we encourage all coauthors to register for an ORCID iD and use it as well. Please encourage your coauthors to verify their ORCID iD within the submission system before final acceptance, as unverified ORCID iDs will not appear in the published article. *Only*  the individual author can complete the verification step; PLOS staff *cannot*  verify ORCID iDs on behalf of authors.

We look forward to receiving your revised manuscript.

Kind regards,

Guichuan Lai

Academic Editor

PLOS One

Journal requirements:

When submitting your revision, we need you to address these additional requirements.

1. Please ensure that your manuscript meets PLOS ONE's style requirements, including those for file naming. The PLOS ONE style templates can be found at

https://journals.plos.org/plosone/s/file?id=wjVg/PLOSOne_formatting_sample_main_body.pdf and

https://journals.plos.org/plosone/s/file?id=ba62/PLOSOne_formatting_sample_title_authors_affiliations.pdf

2. Thank you for your submission to PLOS ONE. Before we can proceed, we kindly ask you to address the following concerns:

We note that you have declared the use of generative AI in the preparation of your manuscript. Please note that contributions by artificial intelligence (AI) tools and technologies to a study or to an article’s contents must be clearly reported in a dedicated section of the Methods, or in the Acknowledgements section for article types lacking a Methods section. This section should include the name(s) of any tools used, a description of how the authors used the tool(s) and evaluated the validity of the tool’s outputs, and a clear statement of which aspects of the study, article contents, data, or supporting files were affected/generated by AI tool usage (https://journals.plos.org/plosone/s/ethical-publishing-practice#loc-artificial-intelligence-tools-and-technologies).

Please therefore revise your manuscript to provide all necessary information to meet these requirements. We appreciate your attention to these queries and look forward to your response.

3. Please note that PLOS One has specific guidelines on code sharing for submissions in which author-generated code underpins the findings in the manuscript. In these cases, all author-generated code must be made available without restrictions upon publication of the work. Please review our guidelines at https://journals.plos.org/plosone/s/materials-and-software-sharing#loc-sharing-code and ensure that your code is shared in a way that follows best practice and facilitates reproducibility and reuse

4. Please note that your Data Availability Statement is currently missing a direct link to access each database. If your manuscript is accepted for publication, you will be asked to provide these details on a very short timeline. We therefore suggest that you provide this information now, though we will not hold up the peer review process if you are unable.

If the reviewer comments include a recommendation to cite specific previously published works, please review and evaluate these publications to determine whether they are relevant and should be cited. There is no requirement to cite these works unless the editor has indicated otherwise.

[Note: HTML markup is below. Please do not edit.]

Reviewers' comments:

Reviewer's Responses to Questions

**Comments to the Author**

1. Is the manuscript technically sound, and do the data support the conclusions?

Reviewer #1: Partly

Reviewer #2: Partly

**********

2. Has the statistical analysis been performed appropriately and rigorously?

Reviewer #1: No

Reviewer #2: Yes

**********

3. Have the authors made all data underlying the findings in their manuscript fully available?

The PLOS Data policy

Reviewer #1: Yes

Reviewer #2: Yes

**********

4. Is the manuscript presented in an intelligible fashion and written in standard English?

Reviewer #1: Yes

Reviewer #2: No

**********

Reviewer #1: This manuscript investigates consistence of host-response transcriptomic associated with bacterial and viral infections. The author proposes a conservative workflow based on modules and their transportability in an external dataset. Overall, the distinction between discovery and external projection is well maintained.

- The main limitation of the study is that the conclusions are based on only one external validation dataset (GSE73461). Include additional datasets or discuss this limitation more explicitly. For example: "These results support the external transportability of selected host-response modules across public infection transcriptomic cohorts." This statement could be slightly softened to avoid overgeneralization.

- The manuscript indicates that Gene Ontology terms were manually reviewed to define modules. More details (criteria) describing how these decisions were made would improve transparency and reproducibility. Are these modules optimal or most informative?

- The manuscript does not compare the proposed strategy with other commonly pathway scoring approaches (GSVA). A brief discussion explaining these limitations should be addressed. Only median differences and adjusted P-values are presented. Include effect size measures and confidence intervals.

Overall, the manuscript addresses a relevant methodological question regarding the reproducibility and transportability of host-response transcriptomic modules across independent datasets. The novelty is methodological rather than biological, regarding the reproducibility of biologically interpretable transcriptomic modules across independent cohorts.

Reviewer #2: SYNOPSIS AND TECHNICAL ISSUES

This manuscript describes a limited and largely sound piece of research seeking to identify characteristic patterns of gene expression that differentiate a viral infection from a bacterial infection in humans. The work explores transcript levels from two published compendiums of transcript information with known disease status. The premise of this work is that there exists collections of genes for which the change in expression reflects the infecting agent and that such collections can be applied more broadly. As I understood the report, one set of data was used as the training set to extract sets of characteristic genes. The expression of these sets of genes was analyzed in a second set of published data to evaluate the discriminatory power of the collection. The authors conclude that it is possible to identify such collections of genes that could be "transported" to other data sets.

The study was limited to these two data sets and did not aggressive explore the power of these particular findings. Specifically, the collections of genes were identified by computational means and with guidance provided to the investigator using information about biological processes (GO identifiers.) The premise was supported by the findings but not challenged. For example, how many of the identified genes were essential for each collection to provide discrimination? This would have been a valuable computational exercise (leave one out, leave two out, etc.) that could extend the utility of the genes identified in this single training set.

The singularly significant finding reported in the Discussion was that discrete programs of gene expression could be found with the power to discriminate among the infecting agent. Viral infections were characterized by antiviral pathways, interferon-stimulated pathways and the more vague anti-viral program of gene expression. Bacterial disease appeared to have a curious fingerprint in translational and ribosome metabolism along with mitochondrial respiration and oxidative phosphorylation.

This finding may prove to be of greater significance in future studies that violate the "directionally" found in these studies. For example, a novel viral disease that presents with the pattern of the bacterial fingerprint may provide unexpected insight into a novel nature of the viral disease and vice-versa. It is for this reason I find the study to be valuable. However, there are additional significant limitations in the manner in which the work was presented.

Technical limitations in this manuscript that need to be corrected include the following:

1. As noted above, the extent to which the gene sets can suffer the loss of specific members and still provide discrimination should be evaluated. Alternatively, if my understanding is so badly off-base, the manuscript should be re-written in a manner to make the objectives more clear to a naïve reader such as myself.

2. The gene sets analyzed were only identified by the accession number (GSE211567 and GSE73461). The nature of the samples needs to be described in substantial detail for independent evaluation by the reader. The content and nature of the gene sets matter much more than the accession number.

3. What was the logic of excluding control samples in the z-score reference set (lines 118-119). Specifically, what was the expected outcome of this exercise and how could it have affected the results?

4. The figure legends (captions) are entirely inadequate because they fail to explain what is depicted in each figure. These legends should state the finding, succinctly describe the method of analysis, and describe the elements in the figure needed to ascertain the finding.

5. What is the formal definition of concordance used here as shown in Fig. 1B?

6. What is the formal definition and mode of calculation for the "module score" from the mean z-score. What is this mean value derived from?

7. What is shown in Fig. 2C? Because the x-axis is not a continuum of values, the y-values have little reason to be joined and expressed in a scatter plot as shown. Moreover, I am a bit unsure of how one defines "projection significance" and what is meant by the "Main projection and "Primary-only z-score sensitivity?

STATISTICS

It is difficult to evaluate the statistical analyses because the methods were not adequately (or clearly) described. In so far as the primary query was a z-score, the results appear appropriate analyzed using corrections for multiple comparisons.

CLARITY

The manuscript is very difficult to understand because of the overly generous use of jargon and phrases that have minimal informative content. I would strongly encourage the authors to write in a simpler style. Even the word "transportable" leans toward jargon. This word, in the context of bioinformatic analyses, should be defined in plain English before being used so heavily.

Other phrases that are unnecessarily confusing include: "coherent expected-direction behavior," "site-aware discovery," "safeguards" (in the context of the analysis of gene expression), "strict discovery/projection firewall," and "frozen," to highlight phrases from the Introduction that could better be conveyed in simple English.

Of particular importance is the notion of "projection." The focus of this paper seems to be that clusters of related genes can be identified and applied to a new dataset to discriminate between viral and bacterial disease without redefining (or rediscovering) the clusters of related genes. This method of "fixed-module projection" should be defined in plain English to reach the broader audience that is typical for the PLoS journals.

**********

what does this mean?). If published, this will include your full peer review and any attached files.

If you choose “no”, your identity will remain anonymous but your review may still be made public.

**Do you want your identity to be public for this peer review?**  For information about this choice, including consent withdrawal, please see our Privacy Policy

Reviewer #1: No

Reviewer #2: No

**********

[NOTE: If reviewer comments were submitted as an attachment file, they will be attached to this email and accessible via the submission site. Please log into your account, locate the manuscript record, and check for the action link "View Attachments". If this link does not appear, there are no attachment files.]

To ensure your figures meet our technical requirements, please review our figure guidelines: https://journals.plos.org/plosone/s/figures

You may also use PLOS’s free figure tool, NAAS, to help you prepare publication quality figures: https://journals.plos.org/plosone/s/figures#loc-tools-for-figure-preparation.

NAAS will assess whether your figures meet our technical requirements by comparing each figure against our figure specifications.

# Author comment: Author response to Decision Letter 1

**DOI:** 10.1371/journal.pone.0353559.r002

10 Aug 2026

Dear Academic Editor and Reviewers,

Thank you for the careful evaluation of the manuscript and for the constructive recommendations. The manuscript has been revised substantially in response. The principal changes include addition of a second accession-level and cross-platform projection cohort (GSE72810), harmonised effect-size and confidence-interval reporting, GSVA sensitivity analysis, exhaustive leave-one/two-gene robustness analysis, expanded cohort and statistical-method descriptions, a revised categorical Figure 2C, clearer definitions and simpler terminology, direct public-data links, public code availability, and a dedicated generative-AI disclosure within Methods.

Each comment is reproduced below, followed by the response and the location of the corresponding revision. The revised submission includes separate clean and marked-up manuscript files.

Academic Editor / Journal Requirements

J1. PLOS ONE style and revision-package requirements

Comment: Ensure PLOS ONE style requirements and file naming are followed.

Response: Thank you. The revision package has now been prepared in PLOS ONE submission form. Separate clean and marked-up manuscript files have been generated from the same locked scientific source and subjected to structural, rendering and visual quality-control checks. Continuous line numbering is retained, the two wide tables remain editable and readable in landscape sections, and the marked-up file uses genuine tracked revisions while accepting those revisions reproduces the clean manuscript content.

Changes in the revised manuscript: Final revision package: PONE-D-26-30583_clean_revised_manuscript.docx and PONE-D-26-30583_marked_up_revised_manuscript.docx.

Supporting analysis/material: Clean manuscript and marked-up manuscript passed the final DOCX structural and visual QA gates.

J2. Generative-AI disclosure

Comment: Provide a dedicated generative-AI disclosure naming the tool, describing use, validation of outputs, and affected study/article materials.

Response: The manuscript now contains a dedicated generative-AI disclosure within the Methods section. The disclosure identifies ChatGPT as an AI-assisted tool provided by OpenAI, describes its use for editorial organisation, language refinement, workflow planning, code drafting and checking, formatting checks and preparation of submission-support materials, states that AI-generated suggestions were not treated as scientific evidence, describes verification of numerical and graphical outputs, and states that the author reviewed the analysis, references, interpretation, figures, tables and manuscript and takes responsibility for the final work.

Changes in the revised manuscript: Methods, 'Declaration of generative AI and AI-assisted technologies in the manuscript preparation process', immediately before the main Results section.

Supporting analysis/material: The declaration wording was preserved during relocation into Methods; clean and marked-up DOCX copies were synchronised and visually checked.

J3. Public availability of author-generated code

Comment: Make author-generated code publicly available without restriction.

Response: The author-generated analysis code and reproducibility materials are publicly available without access restriction in the project repository. The revision branch contains the revision-round scripts, decision logs, quality gates, source-data tables, manuscript-facing figures and supplementary outputs used to support the revised analyses.

Changes in the revised manuscript: Code availability section.

Supporting analysis/material: Public repository: https://github.com/rmaghembe1/host-response-module-transportability; revision branch: https://github.com/rmaghembe1/host-response-module-transportability/tree/plosone_revision_round1_2026.

J4. Direct database links in Data Availability

Comment: Provide direct links to each database in the Data Availability Statement.

Response: The Data Availability Statement has been revised to provide direct NCBI Gene Expression Omnibus links for each of the three principal datasets: GSE211567, GSE73461 and GSE72810. The text also identifies candidate or technical-rehearsal datasets considered during workflow development.

Changes in the revised manuscript: Data availability section.

Supporting analysis/material: Direct GEO accession URLs for GSE211567, GSE73461 and GSE72810 are included in the revised manuscript.

Reviewer 1

Reviewer 1, Comment 1 — Additional external cohort and limitation

Comment: The conclusions relied on one external validation dataset; include additional datasets or discuss/soften this limitation.

Response: We agree that reliance on a single projection cohort was an important limitation. We therefore added GSE72810 as a second accession-level and sample-level whole-blood cohort measured on a different Illumina platform (GPL6947). The cohort contains 146 samples; the prespecified primary contrast comprises 23 definite bacterial and 28 definite viral samples. All five modules retained their expected directions. BACT_M2, VIR_M1a, VIR_M1b and VIR_M2 had confidence intervals excluding zero and passed false-discovery-rate correction in both GSE73461 and GSE72810, whereas BACT_M1 remained directionally concordant but borderline in both. We also softened the replication language because the two GEO accession sets were disjoint and used different array platforms, but direct participant overlap could not be assessed and the studies arose from the same broad investigator network.

Changes in the revised manuscript: Methods: 'GSE72810 cross-platform validation and probe selection' and 'Cross-cohort interpretation and reproducibility boundaries'; Results: 'GSE72810 provides accession-level and cross-platform validation' and 'Cross-cohort effects support four modules in both cohorts'; Discussion limitations.

Supporting analysis/material: Figure 3; Table 2; Supplementary Tables S6-S9; GSE72810 primary Hodges-Lehmann shifts and bootstrap confidence intervals.

Reviewer 1, Comment 2 — Manual GO review and module definition

Comment: Provide more detail on criteria used during manual GO review/module definition and clarify whether the modules are optimal or most informative.

Response: We expanded the Methods to make the module-definition procedure more transparent. Directionally supported discovery features were mapped to genes, assessed by Gene Ontology biological-process enrichment, and reviewed after grouping overlapping or redundant enriched terms. Candidate programmes were evaluated using the documented biological curation hierarchy. We now state explicitly that this was a biologically guided, reproducible curation step rather than mathematical optimisation for group separation or prediction accuracy. External-cohort results were not used to choose or modify module genes.

Changes in the revised manuscript: Methods, 'GSE211567 discovery and module definition'.

Supporting analysis/material: Locked module definitions are unchanged; the revision clarifies the selection rationale and its non-optimisation character.

Reviewer 1, Comment 3 — Comparison with GSVA

Comment: Compare the proposed scoring strategy with GSVA.

Response: We added a GSVA sensitivity analysis in GSE73461 using the same unchanged module gene sets and both z-reference populations. BACT_M2, VIR_M1a and VIR_M1b retained statistical support under both mean-z and GSVA scoring. BACT_M1 was borderline under mean-z scoring but gained statistical support under GSVA. VIR_M2 retained its expected viral-higher direction but became near-zero and statistically unsupported under GSVA. The revision therefore distinguishes direction preservation from method-independent inferential support and describes VIR_M2 as scoring-method-sensitive.

Changes in the revised manuscript: Methods, 'Sensitivity and robustness analyses'; Results, 'Sensitivity analyses retain direction but identify method-dependent support'; Discussion.

Supporting analysis/material: S1 Fig panel B and Supplementary Table S10.

Reviewer 1, Comment 4 — Effect sizes and confidence intervals

Comment: Report effect sizes and confidence intervals in addition to medians and adjusted P values.

Response: We added effect-size estimates and uncertainty intervals throughout the external-cohort analyses. The revised Methods specify Hodges-Lehmann location shifts, rank-biserial effects and stratified 10,000-replicate bootstrap 95% confidence intervals. Figure 3 and Table 2 provide harmonised cross-cohort Hodges-Lehmann effects and 95% confidence intervals for all five modules. This allows the magnitude and uncertainty of the observed effects to be assessed separately from adjusted P values.

Changes in the revised manuscript: Methods, 'Module scoring and statistical analysis'; Results, GSE72810 and cross-cohort effect sections; Figure 3; Table 2.

Supporting analysis/material: Supplementary Tables S8-S9 and cross-cohort Figure 3 source data.

Reviewer 2

Reviewer 2, Comment 1 — Leave-one/two-gene robustness

Comment: Evaluate how loss of specific genes affects module discrimination, for example leave-one-out and leave-two-out analyses.

Response: We performed exhaustive leave-one-gene and leave-two-gene sensitivity analyses for every module under both GSE73461 scoring-reference populations. In total, 29,826 module variants were evaluated. Every variant retained the expected module direction. The minimum Pearson correlation between a deletion variant and its corresponding complete module score was 0.9940, and the minimum Spearman correlation was 0.9897. These results support a distributed module signal rather than dependence on removal-sensitive individual genes or gene pairs.

Changes in the revised manuscript: Methods, 'Sensitivity and robustness analyses'; Results, 'Exhaustive deletion analysis supports distributed module signal'.

Supporting analysis/material: S1 Fig panel C; Supplementary Table S10, including complete leave-one/two-gene results.

Reviewer 2, Comment 2 — Dataset description

Comment: Describe the nature and sample composition of the analyzed datasets in substantially greater detail.

Response: The dataset descriptions have been substantially expanded. GSE211567 is identified as whole-blood transcriptomic data from Sri Lanka and the United States, with a prespecified discovery set of 224 samples (101 bacterial and 123 viral). GSE73461 is described with its GPL10558 platform, 52 definite bacterial, 94 definite viral and 55 control samples in the main z-score reference population, and the restricted primary inferential contrast. GSE72810 is described as 146 paediatric whole-blood samples on GPL6947, including definite, probable, control and uncertain groups and the prespecified 23-versus-28 primary contrast.

Changes in the revised manuscript: Methods, 'Study design and datasets', 'GSE211567 discovery and module definition', 'GSE73461 formal external projection', and 'GSE72810 cross-platform validation and probe selection'.

Supporting analysis/material: Supplementary cohort-audit and sample-classification tables.

Reviewer 2, Comment 3 — Control samples and z-score reference

Comment: Explain the logic and consequences of control-sample inclusion/exclusion in the z-score reference population.

Response: We clarified the role of control and non-primary samples in score standardisation versus inference. In GSE73461, the 52 definite bacterial, 94 definite viral and 55 control samples constitute the main gene-wise z-score reference population, but control samples are not included in the bacterial-versus-viral inferential test. A primary-only z-reference sensitivity analysis quantifies dependence on that choice. In GSE72810, all 146 samples contribute to the main z-score reference population, whereas the primary inferential contrast remains restricted to the 23 definite bacterial and 28 definite viral samples. Additional case-definition and probe-collapse analyses test the robustness of these choices.

Changes in the revised manuscript: Methods, 'GSE73461 formal external projection', 'GSE72810 cross-platform validation and probe selection', and 'Sensitivity and robustness analyses'.

Supporting analysis/material: Figure 2, S1 Fig panel A and the supplementary sensitivity tables.

Reviewer 2, Comment 4 — Figure captions

Comment: Rewrite figure captions so they explain the finding, analysis and figure elements.

Response: All principal figure legends were rewritten so that they explain both the analysis and the graphical elements. The revised legends define direction conventions, sample groups, symbols, confidence intervals or adjusted-P-value thresholds where applicable, and state the principal finding represented in each panel. The S1 Fig legend likewise explains the z-reference/case-definition/probe-collapse sensitivity analyses, mean-z versus GSVA comparison and exhaustive gene-deletion analysis.

Changes in the revised manuscript: Legends for Figures 1-3 and Supporting Information S1 Fig.

Supporting analysis/material: Revised manuscript figure captions and supporting-information caption.

Reviewer 2, Comment 5 — Formal concordance definition

Comment: Provide the formal definition of concordance used in Figure 1B.

Response: We now define directional concordance formally. For each modelled feature, concordance means agreement in the sign of the bacterial-versus-viral log2 fold change between the two contrasts being compared. Percentage directional concordance is the number of same-sign features divided by the number of compared features and multiplied by 100. Spearman correlations of log2 fold changes are reported separately as a complementary measure of ranked effect-size agreement.

Changes in the revised manuscript: Methods, 'GSE211567 discovery and module definition'; Figure 1B legend.

Supporting analysis/material: The definition now matches the implemented cross-site concordance calculation.

Reviewer 2, Comment 6 — Module-score definition

Comment: Define the module score and explain what the mean z-score represents.

Response: We added the explicit module-scoring equations. For mapped gene g in sample i, expression is standardised within the prespecified reference population as z_gi = (x_gi - mean_g) / SD_g. For a module with K mapped and available genes, the sample-level score is the unweighted arithmetic mean, score_i = (1/K) sum_g z_gi. Thus each available mapped gene contributes equally to the primary score; missing genes are omitted only after module coverage has been documented.

Changes in the revised manuscript: Methods, 'Module scoring and statistical analysis'.

Supporting analysis/material: The explicit equations and interpretation of the mean z-score are now given in the manuscript.

Reviewer 2, Comment 7 — Figure 2C categorical display

Comment: Clarify Figure 2C and remove inappropriate lines connecting categorical module values.

Response: Figure 2C was rebuilt to treat the module categories correctly as independent categorical values. Circles denote the main projection and triangles denote the primary-only z-score sensitivity analysis. The module categories are not connected by lines. The dashed horizontal line marks BH-adjusted P = 0.05. The revised caption explains each symbol and why no connecting line is used.

Changes in the revised manuscript: Figure 2C and Figure 2 legend.

Supporting analysis/material: Revised Figure 2C generated by the reproducible figure script; publication outputs include PNG and editable SVG versions.

Reviewer 2 — Statistical-analysis description

Comment: Improve description of statistical analyses.

Response: The statistical-analysis description has been expanded substantially. The revised Methods specify Wilcoxon rank-sum testing, Benjamini-Hochberg correction across the five modules, the interpretation of positive and negative effects, median bacterial-minus-viral differences, Hodges-Lehmann shifts, rank-biserial effects, 10,000-replicate bootstrap confidence intervals, Pearson and Spearman score correlations, and the interpretive basis for cross-method comparisons in the GSVA sensitivity analysis.

Changes in the revised manuscript: Methods, 'Module scoring and statistical analysis' and 'Sensitivity and robustness analyses'.

Supporting analysis/material: Supplementary statistical outputs provide the corresponding complete module-l

Attachment Submitted filename: Response_R1.docx

# Peer review: Decision Letter 1

**DOI:** 10.1371/journal.pone.0353559.r003

**Authors:** Benjamin Liu

26 Aug 2026

External transportability of bacterial- and viral-associated host-response modules across public transcriptomic cohorts

PONE-D-26-30583R1

Dear Dr. Maghembe,

We’re pleased to inform you that your manuscript has been judged scientifically suitable for publication and will be formally accepted for publication once it meets all outstanding technical requirements.

Within one week, you’ll receive an e-mail detailing the required amendments. When these have been addressed, you’ll receive a formal acceptance letter and your manuscript will be scheduled for publication.

An invoice will be generated when your article is formally accepted. Please note, if your institution has a publishing partnership with PLOS and your article meets the relevant criteria, all or part of your publication costs will be covered. Please make sure your user information is up-to-date by logging into Editorial Manager at Editorial Manager® and clicking the ‘Update My Information' link at the top of the page. For questions related to billing, please contact billing support.

If your institution or institutions have a press office, please notify them about your upcoming paper to help maximize its impact. If they’ll be preparing press materials, please inform our press team as soon as possible -- no later than 48 hours after receiving the formal acceptance. Your manuscript will remain under strict press embargo until 2 pm Eastern Time on the date of publication. For more information, please contact onepress@plos.org.

Kind regards,

Benjamin M. Liu, PhD, D(ABMM), MB(ASCP)

Academic Editor

PLOS One

Additional Editor Comments (optional):

Reviewers' comments:

Reviewer's Responses to Questions

**Comments to the Author**

Reviewer #1: All comments have been addressed

Reviewer #2: All comments have been addressed

**********

2. Is the manuscript technically sound, and do the data support the conclusions?

Reviewer #1: Yes

Reviewer #2: Yes

**********

3. Has the statistical analysis been performed appropriately and rigorously?

Reviewer #1: Yes

Reviewer #2: Yes

**********

4. Have the authors made all data underlying the findings in their manuscript fully available?

The PLOS Data policy

Reviewer #1: Yes

Reviewer #2: Yes

**********

5. Is the manuscript presented in an intelligible fashion and written in standard English?

Reviewer #1: Yes

Reviewer #2: Yes

**********

Reviewer #1: Main concerns have been satisfactorily addressed as the inclusion of GSE72810 and GSVA sensitivity analysis.

A few minor suggestion to interpretation of VIR_M2: consistency of VIR_M2 across cohorts was not supported maintained with GSVA.

Repetition: overstatement the absence of gene reselection, module redefinition, reweighting, or model training.

Reviewer #2: All concerns (of reviewer 2) have been well addressed. The reduction in jargon appears to have made the manuscript more accessible to the non-expert reader.

**********

what does this mean?). If published, this will include your full peer review and any attached files.

If you choose “no”, your identity will remain anonymous but your review may still be made public.

**Do you want your identity to be public for this peer review?**  For information about this choice, including consent withdrawal, please see our Privacy Policy

Reviewer #1: **Yes:** Cezar Rangel Pestana

Reviewer #2: No

**********

# Editor report: Acceptance letter

**DOI:** 10.1371/journal.pone.0353559.r004

**Authors:** Benjamin Liu

PONE-D-26-30583R1

PLOS One

Dear Dr. Maghembe,

I'm pleased to inform you that your manuscript has been deemed suitable for publication in PLOS One. Congratulations! Your manuscript is now being handed over to our production team.

At this stage, our production department will prepare your paper for publication. This includes ensuring the following:

* All references, tables, and figures are properly cited

* All relevant supporting information is included in the manuscript submission,

* There are no issues that prevent the paper from being properly typeset

You will receive further instructions from the production team, including instructions on how to review your proof when it is ready. Please keep in mind that we are working through a large volume of accepted articles, so please give us a few days to review your paper and let you know the next and final steps.

Lastly, if your institution or institutions have a press office, please let them know about your upcoming paper now to help maximize its impact. If they'll be preparing press materials, please inform our press team within the next 48 hours. Your manuscript will remain under strict press embargo until 2 pm Eastern Time on the date of publication. For more information, please contact onepress@plos.org.

You will receive an invoice from PLOS for your publication fee after your manuscript has reached the completed accept phase. If you receive an email requesting payment before acceptance or for any other service, this may be a phishing scheme. Learn how to identify phishing emails and protect your accounts at https://explore.plos.org/phishing.

If we can help with anything else, please email us at customercare@plos.org.

Thank you for submitting your work to PLOS One and supporting open access.

Kind regards,

PLOS One Editorial Office Staff

on behalf of

Dr. Benjamin M. Liu

Academic Editor

PLOS One
